# The Impact of the Covid-19 Pandemic on Stroke Volume

**DOI:** 10.1017/cjn.2020.116

**Published:** 2020-06-04

**Authors:** Christopher R. Pasarikovski, Leodante da Costa

**Affiliations:** Division of Neurosurgery, Department of Surgery, University of Toronto, Toronto, Ontario, Canada; Division of Neurosurgery, Sunnybrook Health Sciences Centre, University of Toronto, Toronto, Ontario, Canada

**Keywords:** Stroke, Covid-19, Pandemic, Thrombectomy

On March 11, 2020, the World Health Organization declared Covid-19 a global pandemic. Nations around the world instituted various measures to help combat the spread of the virus. In Canada, provinces declared states of emergency permitting the closure of all nonessential services, and advised citizens to remain in their homes. The goal of these public health measures was to decrease the incidence of new infections through contact tracing, isolation of exposed/infected individuals, and physical distancing. These measures appear to be working, with several provinces and territories reporting decreased incidence of new infections.

Overall, awareness among the general population about the morbidity and mortality associated with Covid-19 infection seems to be high. Given the focus of government resources, constant media attention, and public interest in the pandemic, other equally important health-care issues may be disregarded. For example, a significant decrease in acute treatments for coronary artery disease has been reported in Spain, and in the USA, there has been a decrease in the number of acute stroke investigations, suggesting a decrease in overall stroke care.^1,2^

Sunnybrook Health Sciences Centre (SHSC) is a provincial stroke center and one of the three regional stroke centers serving the Greater Toronto Area (GTA). Similar to other health-care institutions across Canada, significant changes were implemented at Sunnybrook to deal with the Covid-19 pandemic. However, the Hyperacute Stroke Program is considered an essential service and was not affected directly by these restrictions.^3^ There was no institutional directive to limit service, and to our knowledge, there have been no changes in provincial stroke networks. Our institutional practice did not change with respect to endovascular thrombectomy (EVT) eligibility. One exception is that patients unlikely to be candidates for EVT are managed at their local hospital and are not transferred, and during a Covid-19 outbreak at another regional stroke center in the GTA, EVT candidates were transferred to Sunnybrook.

We perceived a drop in total EVT volume and therefore reviewed our code stroke volume (Figure 1). It appears that the overall code stroke volume has decreased since the Covid-19 pandemic. The decrease in stroke volume appears to be in line with the Italian experience reported by Morelli et al., and mimics the interventional cardiology experience in Spain.^1,4^ While it has been reported that Covid-19 infection may be associated with an increased risk of ischemic stroke and large vessel occlusion in younger patients at our center, we observed the opposite, with the Covid-19 pandemic resulting not only in a decrease in the number of EVTs, but in the number of acute code strokes in general.^5,6^ While this may reflect a natural variation in the number of strokes in the population over time, our assumption is that measures aimed at minimizing exposure to Covid-19 have influenced the decision of patients to seek medical attention. Furthermore, emergency room physicians may filter cases more rigorously during the pandemic, particularly if patients have fever and respiratory symptoms.

**Figure 1:**
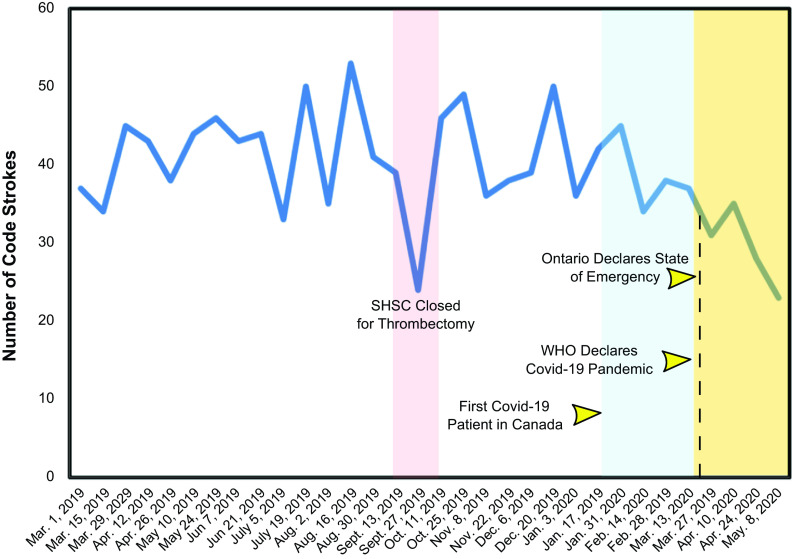
Biweekly code stroke volume from the SHSC from March 1, 2019, to May 8, 2020. Overall, there appears to be a decrease in code stroke volume since the global spread of Covid-19. From the time of the first confirmed Covid-19 case in Canada to the World Health Organization (WHO) declaration of a global pandemic (light blue region), and from the WHO declared pandemic until May 8, 2020 (light yellow region), the stroke volume decrease is appreciated. Note that the decrease in volume in September 2019 was during a time when SHSC was closed for EVT and all potential patients were diverted.

With respect to the various means of patient presentation for code stroke activation (directly via emergency medical services [EMS], transfer from another hospital, emergency room walk-in, and inpatients), it appears that the decrease in code stroke volume is evenly distributed. Between the period of March 1, 2019, and March 16 2020, the number of patients arriving via EMS, transfers from another hospital, emergency room walk-in, and inpatients were 51%, 14%, 23%, and 12%, respectively. In comparison, from March 17, 2020, onward the number of patients arriving via EMS, transfers from another hospital, emergency room walk-in, and inpatients were 59%, 13%, 20%, and 8%, respectively.

Over the past month, we have heard from at least two patients who eventually underwent EVT for intracranial large vessel occlusion that they chose to ignore their initial symptoms (paresthesias in a hemibody) due to concerns of being infected at the hospital emergency room. They only called 911 once the paresthesias progressed to dense hemiparesis. During the same time frame, a patient with aneurysmal subarachnoid hemorrhage was admitted 5 days after ictus. The fear of Covid-19 infection was the reason for not seeking emergent medical care despite severe and persistent headache. At presentation, his neurological condition deteriorated significantly due to dehydration and established symptomatic cerebral vasospasm.

Our analysis is limited to a short time frame, and as mentioned, the changes in stroke volume may reflect natural variations over the year. However, it is reasonable to assume that the number of strokes occurring in the population has not drastically changed, and the concern of being infected with Covid-19 is leading to a delay or refusal to seek medical attention, especially when symptoms at presentation are mild. Since “time is brain,” this may have a negative impact on stroke outcomes. Perhaps a campaign to raise awareness on the negative implications of not seeking medical attention in the setting of stroke symptoms is warranted. The benefits of seeking emergent medical care in case of serious, acute diseases with high morbidity and mortality, such as stroke or myocardial ischemia, far outweigh the risk of Covid-19 infection. As recommended by Heart and Stroke Canada, hospitals are prepared and patients should not delay seeking medical attention if they experience signs or symptoms of stroke.

## References

[1] Rodríguez-Leor O , Cid-Álvarez B , Ojeda S , et al. Impacto de la pandemia de COVID-19 sobre la actividad asistencial en cardiología intervencionista en España. REC Interv Cardiol. 2020;2:82–9.

[2] Kansagra AP , Goyal MS , Hamilton S , Albers GW. Collateral effect of covid-19 on stroke evaluation in the United States. New Engl J Med. 2020:NEJMc2014816. doi: 10.1056/NEJMc2014816 PMC7233187 32383831

[3] Khosravani H , Rajendram P , Notario L , Chapman MG , Menon BK. Protected code stroke: hyperacute stroke management during the coronavirus disease 2019 (COVID-19) pandemic. Stroke. 2020;51(6):1891–5. 32233980 10.1161/STROKEAHA.120.029838 PMC7258750

[4] Morelli N , Rota E , Terracciano C , et al. The baffling case of ischemic stroke disappearance from the casualty department in the COVID-19 Era. Eur Neurol. 2020:1–4. 10.1159/000507666 PMC7179532 32289789

[5] Oxley TJ , Mocco J , Majidi S , et al. Large-vessel stroke as a presenting feature of covid-19 in the young. New Engl J Med. 2020;382:e60 32343504 10.1056/NEJMc2009787 PMC7207073

[6] Moshayedi P , Ryan TE , Mejia LLP , Nour M , Liebeskind DS. Triage of acute ischemic stroke in confirmed COVID-19: large vessel occlusion associated with coronavirus infection. frontiers in neurology. Front Neurol. 2020;11:353. 10.3389/fneur.2020.00353 PMC7186326 32373061

